# A Web-Based Caring Training for Caregivers of Children with Cerebral Palsy: Development and Evaluation

**Published:** 2018

**Authors:** Zahra NOBAKHT, Mehdi RASSAFIANI, Seyed Ali HOSSEINI

**Affiliations:** 1 Pediatric Neurorehabilitation Research Center, University of Social Welfare and Rehabilitation Sciences, Tehran, Iran.; 2 Occupational Therapy Department, Faculty of Allied Health Sciences, Kuwait University, Kuwait.; 3 Pediatric Neurorehabilitation Research Center, University of Social Welfare and Rehabilitation Sciences, Tehran, Iran.; 4 Social Determinants of Health Research Center andOccupational Therapy Department, University of Social Welfare andRehabilitation Sciences, Tehran, Iran.

**Keywords:** Web-based intervention, Cerebral palsy, caregiver

## Abstract

**Objectives:**

Caregivers of children with cerebral palsy (CP) have to spend a long time to take care of their children. We aimed to develop a user-friendly web-based intervention for training parents of children with CP and evaluate the process of development using modified CeHRes roadmap.

**Materials & Methods:**

The study was conducted from September 2016 to September 2017 in Tehran, Iran. We did it in four main steps including determining the needs of users, content development, design, operational development and evaluation.

**Results:**

The website for caregiver training provided nine general topics and had the possibility that the caregivers could determine their educational priorities. Moreover, the users could share their experiences with other users and could ask questions from an expert. Ten caregivers completed a usability questionnaire after four weeks of use. The average score of 70.5 out of 100 was shown among caregivers. The average score of all statements was above three on a Likert scale between 1 and 5.

**Conclusion:**

The website has the possibilities including registering caregivers of children with CP, the possibility to confirm registration with an SMS and the possibility to determine the caregiver educational priorities. It has the usability for training caregivers of children with CP.

## Introduction

Cerebral Palsy (CP) describes a group of permanent disorders of the development and posture, causing activity limitation, attributed to non-progressive disturbances that occurred in the developing fetal or infant brain (1). Children with CP are more dependent on their caregivers to perform their Activities of Daily Living (ADL). As a result, parents of these children have to spend a long time to feed, bathe and clothe a child with the low capability of mobility (2). Therefore, taking care of these children with disability is time-consuming and is a source of stress for their caregivers. Dealing with stressful situations has a negative impact on caregivers’ quality of life. Taking care of a child with CP affects physical and social welfare, freedom and independence, comfort and financial stability of the family (3, 4).

Majority of caregivers of children with CP had a low or moderate level of knowledge about appropriate caring for their children (5). Some caregivers do not receive any training in this area or the training they receive is not commensurate with their educational needs (6). To reduce or prevent their problems, these parents and caregivers are required to receive special training in caring for these children. There is evidence that shows the effectiveness of parental training in increasing parents' knowledge, reducing their stress and improving their quality of life (6-8). There are several methods for caregivers’ training including face to face training, training through workshops, offering booklets, training through videos and using telehealth. The effectiveness of caring training through workshops, offering booklets and face to face training in Iran is documented(7, 9, 10). In the field of telehealth, there is growing evidence of providing web-based interventions for people with diabetes and another diagnosis in Iran (11-14). Internet penetration rate in Iran is more than 50% and increased dramatically in recent years. Internet penetration rate is an indicator that represents the percentage of the population of a country or region that uses the Internet (15).

Telehealth is a broad term that includes both telemedicine and telerehabilitation and refers to the use of electronic information and telecommunication technologies to provide health-related services (16). Telehealth as a model of service delivery can be used in various fields including evaluation (tele-evaluation), intervention (tele-intervention), consultation (teleconsultation), monitoring a client (telemonitoring) and supervision (tele-supervision) (17, 18). Interest in the use of web-based intervention is increasing along with development in information and telecommunication technologies. It is now widely used in various fields including education and counseling for different age groups and a variety of diagnoses. Therefore, this model can be used to provide caring training for caregivers of children with CP. The use of telerehabilitation in comparison with other interventions provides benefits such as better clinical outcomes, more participation, and completion of interventions, more time for consultations and more client satisfaction (19). Accessibility to professionals and avoiding unnecessary delays in receiving care are other positive points about this model (19). Rehabilitation is a long-term and continuous process which sometimes leads to the disruption of a caregiver’s job, daily routines and the role of the family member. Satisfaction of clients with this model is high due to saving their time and low cost of services (19, 20).

Studies with the aim to development and evaluate a web-based intervention are summarized in Appendix 1. There are various methods for development of a web-based intervention and evaluation of the process of development. These include CeHRes roadmap, Intervention Mapping protocol, lifecycle method, etc. the CeHRes Roadmap is one of the methodsused as guidance for the developmental process of web-based interventions (21-23). This roadmap is generally appropriate for telehealth studies in which all the requirements and details needed for development are considered (24). 

This roadmap consists of five main steps as follows: The first step is to investigate the situation. The goal is to determine the problem with health care, what the contribution of technology would be to meet the problem and who will benefit from this technology. Research stakeholders with different backgrounds (financial stakeholders, patients, caregivers, etc.) are identified by the research team. The second step is to determine the value. This step refers to articulating the previous step. The third to fifth steps respectively are design, operationalization, and summative evaluation. Evaluation in this roadmap is done through two methods including 1) formative evaluation, done at each step with the aim of evaluating each step of the process; and 2) summative evaluation, which determines what can be achieved within the specified time. In this roadmap, formative evaluation is emphasized. At the end of each step experts’ and users’ comments are necessary for making necessary changes. Summative evaluations must consider both the uptake and impact of eHealth technologies. Uptake of eHealth technologies refers to the data received from the website, for instance, number of logins. Impact of eHealth technologies denotes the data gathered by outcome measurement (24). Intervention mapping protocol was used in two studies as a general framework (25, 26). 

This is a six-step protocol. 1) Determining the needs of the study population, 2) Determining performance objectives, and change objectives, 3) Determining the methods based on the theory and practical applications, 4) Developing and pretesting program components,5) Adoption and implementation and 6) Evaluation (26). This protocol covers development and intervention but formative evaluation is less pronounced in this protocol in comparison with the CeHRes roadmap. It can be used to design any intervention. Due to the importancegiven to the intervention in IM protocol special emphasis has not been put on the design and providing a model in IM protocol. The life cycle method consists of five stages: 1) Determine the needs of the user, 2) System design, 3) System development, 4) System evaluation and 5) System Application (27). In this method, unlike the IM protocol emphasis is on system design. The content development and attention to technology needs in accordance with user needs are not considered between the first and the second stage. The process of formative evaluation in this method has not received sufficient attention. Therefore, we used CeHRes Roadmap in accordance with our research conditions.

It is essential to provide training for caregivers. Continuous attendance in rehabilitation centers, traveling distances and spending a lot of money make it difficult for a caregiver to care for a child with cerebral palsy. It should consider providing training and childcare facilities in a child's living environment, so they do not have to spend a long time and cost. This issue is important in large cities, small towns and remote areas. In big cities, difficulty intraveling over long distances and in small towns, limited numbers of experts reduce training accessibilities. Therefore, online education is essential. The internet-based health service was theorized usage of family caregivers. They mapped three main factors influenced the use of the intervention: a) caregiver needs (personal capacity, available social support, and caregiving belief); b) information communication technology (ICT) factors (accessibility barriers and perceived efforts to use the technology); and c) style of using the technology (preference for using e-mail or the customized website). New caregivers employed interactive intervention such as using e-mail and more experienced caregivers used more reflective learning such as information on the website(28). Therefore, a web-based intervention for caregivers of children with CP is more appropriate for more experienced caregivers. 

In general, the quality evaluation of the developmental process of a web-based intervention can be done through various procedures including system quality, content quality, and service quality. System quality means the technology is user-friendly, secure and easy to access. Content quality means the content is understandable, meaningful and convincing. Service quality refers to whether the service is provided sufficiently (24, 29). 

This study was conducted with two aims. The first was to develop a user-friendly web-based intervention for training caregivers of children with CP. The second was to evaluate the process of development. To achieve these aims the method of development and evaluation was specified by the researcher and also the factors affecting the researcher’s decision about the method was determined. 

## Materials and Methods

Our study applied steps of the CeHRes Roadmap with some modification in accordance with the research conditions. The method was implemented in four main steps. In the first step (determine the needs of users), caregivers of children with CP were asked about information concerning their training needs. In the second step (content development), content for caregivers web-based training was developed by the experts. In the third step (design), website prototype was designed and in the fourth step (operational and evaluation), a pilot study was conducted.

Approval was obtained by the Ethics Committee of the University of Social Welfare and Rehabilitation Sciences IR.USWR.REC.1395.92. Informed written consent was received from participated caregivers. 

**Determining the needs of users**

This step was performed by one of the authors (M, R) through a separate qualitative content analysis study (30). Fifteen in-depth interviews were conducted with mothers of children with CP who participated in face to face training program. The aim was to seek information on barriers and facilitators of the use of the training by mothers. Inclusion criteria include mothers of children with CP with Gross Motor Function Classification System (GMFCS) (31) level ΙΙΙ, ΙѴ, and Ѵ, aged from 4 to 12 yr. They were recruited from Occupational Therapy Clinics in Tehran, Iran. Mothers attended a workshop and received a booklet and then used training for three months. This period was accompanied by an occupational therapist telephone follow-up. After three months, 15 mothers were interviewed individually. The interviews were recorded and then transcribed. To get more information about this step please see the article (30).

**Content development**

During this step, first a questionnaire -was developed by the research team- was sent by E-mail to a number of experts with the experience of training parents and caregivers of children with CP. Four women and one man, with one of them holding a Ph.D., three being Ph.D. students and one holding a Master with the mean age of 29.6 (SD= 5.46) yr and the mean clinical experience 6.4 (SD= 4.34) yr completed the questionnaire. These researchers had used the content of face to face caring training of children with CP for caregivers in their studies. This questionnaire asked the researcher whether the training topics were helpful or not and are the training topics necessary to be maintained? In this part of the study, two of the researchers received the questionnaire by email and sent it back after completion. The other three completed the questionnaire in a face-to-face meeting. The comments received were discussed in the research team and decisions were made about changes. 

In order to seek information on whether the website homepage has particular importance to attract the attention of caregivers to the necessity of the topic, a questionnaire was completed by the experts who took part in the previous step. After explaining the purpose of this questionnaire, the researchers were requested to offer their comments in two parts, first about some statements presented with images in the website homepage and second about some texts for more information. The received comments were discussed by the research team and decisions were made about necessary changes by expert consensus.

**Design**

A paper prototype was first designed. Then, in a meeting with a website designer the paper prototype was discussed and requirements for the development of the website were determined. Then, a power point prototype was designed and evaluated by the research team. After that, the website was developed by the designer. The website was developed in HTML, jQuery, and bootstrap (9) and the database management system SQL server (2014). The program was written using Asp.net MVC (4).

**Operational and Evaluation**

In this step, the web-based training was applied to 10 caregivers of children with CP for four weeks. We decided on 10 to cover caregivers with variety of education and experience of using the internet. Inclusion criteria were included mothers of children with CP with (GMFCS) (31) level ΙΙΙ, ΙѴ, and Ѵ, aged from 4 to 12 yr. They were recruited from occupational therapy clinics in Tehran with convenient sampling. At first, these caregivers were invited to an individual meeting with the researcher to become familiar with use of the website on either mobile phones or personal computers. After registration and user verification by the administrator, they set their educational priorities and downloaded the first priority in their first meeting. During the meeting, discussions, questions, and problems encountered by the caregivers were recorded by the researcher. Then, caregivers downloaded their next four weeks priorities. After four weeks, the usability of the website was tested on these 10 caregivers using WAMMI (32) questionnaire. The questionnaire consisted of twenty statements with a five-point Likert scale (strongly agree to strongly disagree). The scores of statements in negative terms were reversed. Higher scores reflect greater usability of the website.

## Results

**Determining the needs of users**

Facilitating factors in training caregivers were divided into three groups: Factors related to workshops, booklet and persistent relationship between parents and therapists. Caregivers approved the appropriateness of content with their needs.

Caregivers were more motivated to do home care training program if attention was paid to parents’ educational needs, their physical and mental health and if parental awareness was increased and also if the parents were counterparts in the network of caregivers with similar experience. Due to the importance of educational needs of caregivers, the possibility of determining educational priorities was considered on the website. Moreover, the possibility to communicate and express similar experiences was provided on the website.

**Content development**

**Figure 1:**
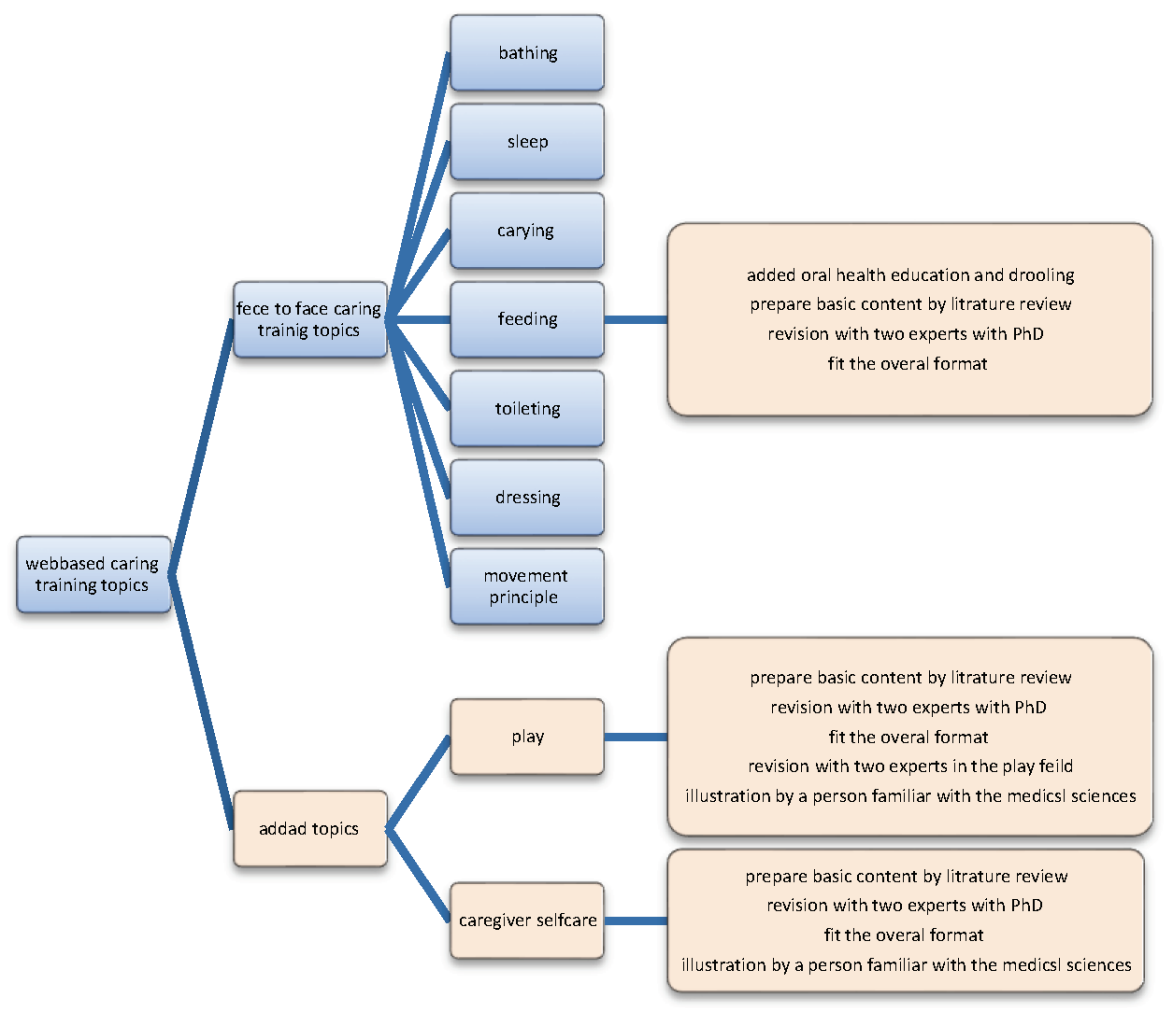
Web-based caring training topics

The summaries of suggestions were discussed by the research team and decisions were made about changes. The topics provided in face to face training were maintained for web-based training. Self-care education (for caregivers) and play were added to the topics. Therefore, web-based training was prepared in nine general topics (Figure 1).According to the experts’ suggestions for greater impact, the training was presented weekly. Training was provided in accordance with the priorities of caregivers. For determining the content of the website homepage, proposed sentences and texts were discussed by the research group and three sentences were selected for presentations to accompany the picture on the homepage including "Do you want to reduce the time it takes to take care of your child?; Do you know after receiving the training program caregiver musculoskeletal pain is reduced?; And do you want your child to be more independent in her/his own care?" Moreover, some texts about CP, motor growth prediction and concepts related to daily care were added to the homepage and user page to enhance the user’s personal information. To develop the required texts, the basic content of the texts were prepared by one of the researchers. Then, two experts with Ph.D. and over 20 yr of experience working with children read the prepared texts and presented their suggestions and made corrections.

**Design**

In the first part, the paper prototype was designed and analyzed with a web designer. Some facilities were considered for the user. These facilities included having a personal page, possibility to determine training priorities based on needs, communication among members and the possibility of questions and answers with an expert. Some facilities were considered for the administrator. These facilities included user verification, answers to questions; report lists of the users, number of entries and downloads, transferring Frequently Asked Questions to the homepage and the potential of providing new information.

In the second part, with the purpose of visualizing ideas, a PowerPoint prototype was designed. In this stage, all content put on the website prepared in the previous steps and information necessary to subscribe (approved by the group of experts) was inserted into a PowerPoint prototype with some pictures for visual attraction. After verification by the research team, the prototype was presented to the web designer to start website development. 

**Table 1:** Demographic information of participants in the usability test

Distribution of participants		
Caregiver age (year)		
	Mean (sd)	32.6 (5.72)
Caregiver education		
	< diploma	3
	diploma	2
	bachelor	4
	master	1
Caregiver experience of use of internet		
	low	3
	moderate	5
	alot	2
Child age (month)		
	Mean (sd)	80.03 (29.26)
Child sex		
	female	4
	male	6
MACS level		
	Level ΙΙ	1
	Level ΙΙΙ	3
	Level ΙѴ	3
	Level Ѵ	3
GMFCS level		
	Level ΙΙΙ	3
	Level ΙѴ	3
	Level Ѵ	4

**Figure 2:**
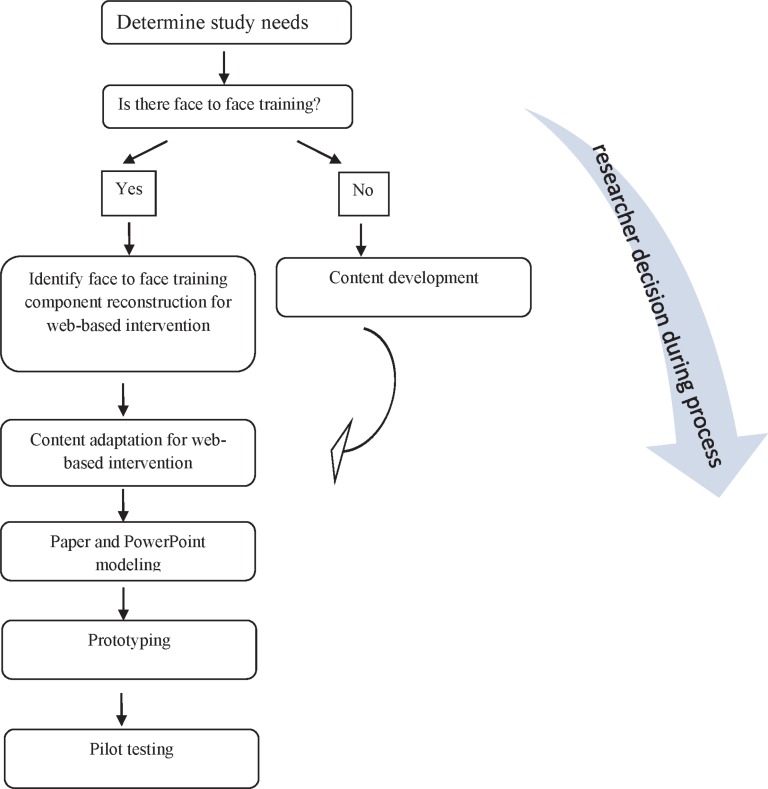
Researcher flowchart for designing web-based interventions

**Appendix 1:** Summaries of studies with the aim to development and evaluate a web-based intervention

**Search strategy:** **Databases: Pubmed, OVID, ProQuest, Web of science, Elsevier, OT seeker, SID, Magiran, IRAN MEDEX, MEDLIB and Google scholar** **Keywords: (development, standardization),(validity, reliability, pilot testing, evaluation, formative evaluation, summative evaluation) and (web-based intervention, web-based program, teleintervention)**			
Author	Goal	Framework	Methods
Skjoth 2015	Development of a web-based decision aid for participation in down syndrome screening	CeHRes roadmap	Determine the executive team consists of research group, developers and expert group Search the database for background information Interviews with professionals and pregnant women Field observations Design model according to the comments Evaluation by two experts and six pregnant women
Ramadas 2015	Web-based dietary intervention for people with type 2 diabetes	Transtheoretical model's stages of change and user-centered design approach	Review literature and guideline by research panel includes a nutritionist, behavioral psychologist, public health specialist, endocrinologist and epidemiologist Prepare twelve lesson plans in a intervention package according to the regime change process for change attitudes, knowledge and behavior in relation with diet Pilot study to evaluate the acceptability and user-friendliness of the intervention (n=30) Paper prototype Mock prototype Alpha testing web details by webmaster Beta testing to assess acceptability and user-friendliness web design (n=30)
Poelman 2013	Development and evaluation ofinternet-based interventions to raise awareness of food portion sizes	Transtheoretical model	Provide content based on relevant text Content compatibility with transtheoretical model Observation by eight experts in the prevention of obesity Pilot study (n=5)
Lee 2013	Development and evaluation web-based self management training and dietary intervention program for the cancer survivors	Life-cycle method	Determine the needs of users with relevant literature review and interviews with semi-structured questions with cancer survivors and specifying system function requirements with reviewing of other health web-based management programs for cancer survivors and trans-theoretical model strategies such as stage-matched education and feedback System design System development Usability and accuracy evaluation of the content in a group of experts including nutritionist, exercise physiologist, nurse, web designer, web developer. Ease of use evaluation by questionnaire (n=29 breast cancer survivor)
Kelders 2013	Development of web-based intervention to prevent depression	CeHRes Roadmap	Contextual inquiry with literature review Value specification with semi-structured interviews with people who had mild depressive symptoms (n=18) rapid prototyping simultaneously and meeting the research team Design with the evaluation by experts and users
Heckman 2015	Development of Internet intervention to demonstrate behaviors associated with risk of skin cancer in young adults.		Planning the intervention: interviews with people participated in face to face training (n=25) and focus group interviews to shape content and web application Web content development: composition of the content resulting from texts and interviews, avatar development and content understandability evaluation by the experts Preliminary assessment and revision: cognitive interview, acceptability testing with structured interviews, usability testing by questionnaire and interview, quality control testing Pilot testing: clinical trial (n=53)
Bravender 2013	Web-based intervention Development and use it to enhance the relationship between doctor and adolescents about healthy weights	Social Cognitive Theory Physician-barrier model	Intervention content and moves development by expert teams Content revision and edition by four pediatric primary care professionals Audio clips preparation based on basic information and their code Website design Send web address and username and code with an email Meet face to face and explain intervention
Danaher 2012	Development and process evaluation web-based training program of responsible beverage service		Programdevelopment includes the design of each program module, its function, information architecture and instructional design Focus group interviews with curators, managers and employees to determine encouraging factors to use this program and feedback about the type and presentation of content in alpha sample (n=9) Usability evaluation by think aloud modeling techniques in beta sample (n=3, 7) Implementation (n=112)
Fledderus 2015	Development and evaluation online Relapse-prevention program based acceptance and commitment therapy for patients with chronic pain	CeHRes roadmap	Contextual inquiry: focus group session with patients with chronic pain (n=10) and researchers (n=2) Value specification: based on the needs identified in the previous step. Design a prototype for a website and some prototypes for mobile application with powerpoint. Then presented to participants (n=28) and they were interviewed with semi-structured interviews about usability and clarity of prototypes. Design of the technology: design were based on the previous steps information. Usability evaluation was done with think aloud modeling technique from user (n=5) and expert (n=9). Pilot study for two months (n=17), telephone interview about the helpfulness of the program
Ghahari 2009	Development, standardize and pilot study of online fatigue self-management program	Problem solving	Identify the basic components of face-to-face training for online program with free discussion (deconstruction) Design a prototype and model (reconstruction) Formative evaluation in three pilot stage.
Dew 2004	Develop and evaluate web-based intervention to improve the psychological consequences in heart transplant recipients and family caregivers	Problem solving	Content development with the literature review and focus groups Web-based intervention for 4 months Accessibility and user satisfaction evaluation with using information from website and question from users
Springvloet 2014	Development and evaluation protocol of two versions of Web-based nutrition training intervention for adults with cognitive and environment feedback	Intervention mapping protocol	Needs assessment, determine what is the needs of the study Determine performance objectives, and change objectives Determine methods based on theory and practical applications Development of online intervention: use available commuter version, consumer panel (n=55), pre-test and understandability and ease of use evaluation (n=44)
Gelatt 2010	Development and evaluation of interactive web-based program for step families		
Walters 2014	Development of web-based intervention for substance abuse treatment in criminal justice system	Extended parallel process model Motivational interviewing Social Cognitive Theory	Content development in the form of questions and answers Provide audio feedback Initial assessment (n=21) in the form of application and content
Willems 2015	Systematic development of web-based intervention providing psychological and life style support for cancer survivors	intervention Mapping protocol Problem Solving cognitive behavioral therapy	Determine the needs, reviewing the literature, focus group interviews and survey Determine performance objectives, and change objectives Determine methods based on theory and practical applications Developing program components Approval and implementation Assessment
Martorella 2013	Development and validity of virtual nursing intervention to improve self-management of pain after heart surgery	A nursery model	Determine the clinical problem Design general view Clinical Operations Production Pilot study (n=30)

Website development was conducted with multiple checks by the research group. In the first modification, website animated images became smaller with more emphasis on their texts by resizing and changing their font color. In the second modification, for explaining the purpose some texts were added to the homepage regarding what is designed, who are the targets groups of the website, what services will be offered on the website and how to use these services. For this purpose, a text was prepared and approved by two experts. In the third modification, children's drawings were used instead of images for the purpose of offering native pictures. Besides, short message service was added to the website to for user verification and to remind the user to download the next priority. The possibility to communicate with similar caregivers and professionals was provided. The accuracy of these conversations was controlled by administration (an Occupational Therapy Ph.D. student) and an expert with Ph.D. The guide for the website was developed. After multiple tests by the designer and the research team, the website was ready for use in the next step (http://www.cpcare.ir). All stages of development all web design standards were noted (33).

**Operational and Evaluation**

Participants' demographic information is shown in Table 1. In this step, questions asked by the caregivers or difficulties faced by them during the first meeting were summarized and discussed by the research team. For example, pages whose font size was said to be small or whose image size was thought to be large were resized by the designer. Or where the caregivers had to spend time to find the buttons, their access was increased. At this stage, the explanations on the register page were shortened to reduce the time required to complete the registration process. Moreover, to increase the speed and ease of use some explanations were added to the website guide. After completion, the usability questionnaire the average score of 70.5 was observed among caregivers. The average score of all statements was above three.

## Discussion

This study aimed to develop web-based intervention for daily care training for caregivers of children with CP and evaluate the process of its development. At first, for decision making on how to develop and evaluate web-based intervention, the researcher reviewed some studies mentioned in Appendix 1. After summarizing the various methods used in the studies for web-based development and evaluation, a method was specified. This method was modified according to different factors including access, finance and time. The process of the development and evaluation was a dynamic process and needed researcher’s decision making during the process. In this section, the process is discussed with regard to other studies reviewed in Appendix 1 to determine the factors affecting researcher’s decision-making.

In accordance with research factors that influence researcher’s decision, CeHRes Roadmap was used with some changes. For example, the first two steps of this method in our study were conducted as a separate study to determine the user's needs. In the second step, the content was developed. Next steps were based on the roadmap.

In our study, the first step was to determine the user's needdone in a separate qualitative study. Mothers mentioned that they will be motivated if more attention is paid to their educational needs then in our study the possibility of determining educational priorities was considered on the website. In addition, they wanted to have networked caregivers with similar experiences the possibility to communicate and express similar experiences was provided on the website. They approved the appropriateness of content with their needs. The effectiveness of the face to face content was also approved in studies (7-9, 34). The step "determine the needs of the user" has been conducted as a first step in developing a web-based intervention study(22, 23, 25-27, 35). This step was done followed reviewing relevant literature and interviews with the users. Content and related technology requirements in accordance with user needs were developed in the next step. However, some studies have reported this as a first step (27, 36-40). Considering that the content in of face to face daily care training of children with CP for caregivers was available, and its effectiveness has been examined in various studies, in our study the first step was to determine the user's needs. After that, face to face training content was developed for web-based intervention. Performing a step like determining user requirements or contextual inquiry increases contact between the researcher and users which is a necessary component. The results of this step led to the better use of technology to meet the user's needs. In our study caregivers had the possibility to communicate with caregivers with similar experiences to facilitate greater use of training. If a researcher does not have access to face to face or web-based training, content development must be done before the design. In our study, there was access to face to face training. So it was improved for use in web-based training. All face to face training topics were maintained and some topics were added to them. According to the caregivers' and experts' views, training was provided weekly. It was essential to take these two steps prior to the design phase to consider the website requirements.

In our study modeling and analysis were performed by multiple models with paper, PowerPoint, and prototype. Use of paper and PowerPoint and prototype modeling can be quite helpful because Web-based intervention development in the early stages is abstract. Design was done in the next stages of the studies. Designing a model has been done with paper, PowerPoint or prototype model and then feedbacks from users were received for editing (21, 41, 42). These feedbacks were considered as formative evaluation. Model designing improves evaluation procedures and can help to reduce the time needed for design. 

In our study, considering research conditions, evaluation was conducted in two stages including implementation and getting feedback from users and usability evaluation. For evaluating the process of web-based intervention studies have used various methods including assessing acceptability and website usability through a pilot study and getting feedback from users (41), assessing the usability and accuracy of the content in the group of experts, and user-friendliness with completing questionnaires by the user (27), content understandability assessment by experts, acceptability assessment with the structured interviews, usability evaluation by questionnaires and interviews (37), usability evaluation via think aloud modeling technique (43), usability and clarity evaluation with semi-structured interviews and usability evaluation through think aloud modeling technique (23) and accessibility and user satisfaction evaluation using information from website (36). Thus evaluations were done in terms of accuracy, intelligibility, and clarity of content as well as design usability and user satisfaction. The evaluations have been conducted in various ways including interviews, cognitive techniques; think aloud modeling technique, completing questionnaires and meetings with users and experts. Data were also obtained from the website. In our study, after first session implementation feedbacks from users received and after four weeks usability evaluation was done. It shows that our website has acceptable usability and it is easy to use by mothers with different level of education and internet experience.

The process of the web-based intervention development is a dynamic process. In our research, we had to add some possibilities to match the needs faced during development. For instance, users of our website had the possibility to ask their questions of an expert. Hence, when the expert answered their questions, they were not aware of the response. The research team decided to incorporate short message service (SMS) module into the program. The users received an SMS informing that the expert answered their questions. Moreover, this service was used to remember the user to download the next priority and user verification by the system. 

The website has the possibilities including registering caregivers of children with CP with the registry form including GMFCS, Manual Ability Classification System (MACS) (44) (was recorded according to the parent questionnaire in website) and IQ level (SPARCLE study)(45), the possibility to confirm registration with an SMS, the possibility to determine the caregiver educational priorities and to remind the caregiver to download priority weekly. The users can share her/his experiences with other users and can ask questions of an expert. The effectiveness of the web-based caring training for caregivers of children with CP must be determined in a randomized controlled trial. We would also suggest assessing parental satisfaction as well as the impact it has on the children.

In the present study, it was not possible to take into account the level of caregiver experiences. Therefore, it is suggested that to pay attention to the amount of caregiver experience in future studies. Effective factors were identified in the use of Internet-based services. These factors included caregivers' needs, factors relating to communication technology, and information use style. Theexperience of caregiver affects the style of using that information (28). 

In Iran, the majority of caregivers of children with cerebral palsy are mothers; we provided our training to mothers as the main caregiver. Future studies are presented to the father and other family members.

**Limitations**

This type of research requires multiple investigations performed by the user at different stages. In this study due to time limited access to users at some steps was limited. It seems better to follow each step in a separate study to spend enough time on every step.

Most of caregivers of children with CP in the study context were mothers. Then caregivers in this study were mothers. This might limit the generalizability of study results to fathers who are also caregivers. 

One of the limitations we encountered during the study was the speed of the internet and its interruptions, which user had to reconnect.

**In conclusion, **for web-based intervention development, it seems more effective to first determine study needs and then proceed with content development. If face to face intervention is available, it can speed up the design process. The main components of face to face intervention could reconstruct for web-based intervention. If there is no access to face to face intervention, content development can be conducted based on the literature reviews and experts’ and users’ views depending on the objectives of interventions and theories and methods. Then content adaption must be done by specifying technical requirements and methods for web-based content. In the third step, designing paper or PowerPoint models and receiving users’ and experts' comments and designing a prototype model and initial testing can improve the process. Finally, preliminary testing seems to be helpful for summative evaluation (Figure 2). To develop and evaluate a web-based intervention access is essential including access to various experts including website design experts and experts familiar with content, hardware and software accessibility, access to the same people supposed to use certain websites, access to finance. In addition, lack of time that affects the researcher’s decision during the research process.

Our website has the possibilities including registering caregivers of children with CP, the possibility to confirm registration with an SMS and the possibility to determine the caregiver educational priorities and also has the usability for training caregivers of children with CP.
